# Steering of Vortices by Magnetic Field Tilting in Open Superconductor Nanotubes

**DOI:** 10.3390/nano14050420

**Published:** 2024-02-25

**Authors:** Igor Bogush, Vladimir M. Fomin, Oleksandr V. Dobrovolskiy

**Affiliations:** 1 Leibniz IFW Dresden, Institute for Emerging Electronic Technologies, Helmholtzstraße 20, 01069 Dresden, Germanyv.fomin@ifw-dresden.de (V.M.F.); 2 Moldova State University, Faculty of Physics and Engineering, Str. A. Mateevici 60, 2009 Chişinău, Moldova; 3 University of Vienna, Faculty of Physics, Nanomagnetism and Magnonics, Superconductivity and Spintronics Laboratory, Währinger Str. 17, 1090 Vienna, Austria

**Keywords:** superconductivity, 3D nanostructures, vortex dynamics, microwave frequencies

## Abstract

In planar superconductor thin films, the places of nucleation and arrangements of moving vortices are determined by structural defects. However, various applications of superconductors require reconfigurable steering of fluxons, which is hard to realize with geometrically predefined vortex pinning landscapes. Here, on the basis of the time-dependent Ginzburg–Landau equation, we present an approach for the steering of vortex chains and vortex jets in superconductor nanotubes containing a slit. The idea is based on the tilting of the magnetic field B at an angle α in the plane perpendicular to the axis of a nanotube carrying an azimuthal transport current. Namely, while at $\alpha =0^{\circ }$, vortices move paraxially in opposite directions within each half-tube; an increase in α displaces the areas with the close-to-maximum normal component $|B_{n}|$ to the close(opposite)-to-slit regions, giving rise to descending (ascending) branches in the induced-voltage frequency spectrum $f_{U}\left(\alpha \right)$. At lower *B* values, upon reaching the critical angle $\alpha_{c}$, the close-to-slit vortex chains disappear, yielding $f_{U}$ of the $nf_{1}$ type (n≥1: an integer; $f_{1}$: the vortex nucleation frequency). At higher *B* values, $f_{U}$ is largely blurry because of multifurcations of vortex trajectories, leading to the coexistence of a vortex jet with two vortex chains at $\alpha =90^{\circ }$. In addition to prospects for the tuning of GHz-frequency spectra and the steering of vortices as information bits, our findings lay the foundation for on-demand tuning of vortex arrangements in 3D superconductor membranes in tilted magnetic fields.

## 1. Introduction

Knowledge of how magnetic flux quanta (Abrikosov vortices) move and arrange themselves under various currents and magnetic fields is critical for supercurrent flow and fluxonic applications. For instance, while for defect-free planar thin films, a hexagonal vortex lattice [1] is expected, defects and the sample geometry make vortex patterns differ from a regular lattice [2,3,4]. In planar thin films, the places of nucleation of vortices are determined by edge defects [5,6], current-crowding effects [7,8,9] or their combination [10,11]. For structures with perfect edges, a single edge defect acts as a local injector of vortices [12]. Driven by competing current–vortex and vortex–vortex interactions, such vortices form a jet, which is narrow at the defect and *expands* due to the repulsion of vortices as they move to the opposite edge of the structure [13].

**Figure 1:**
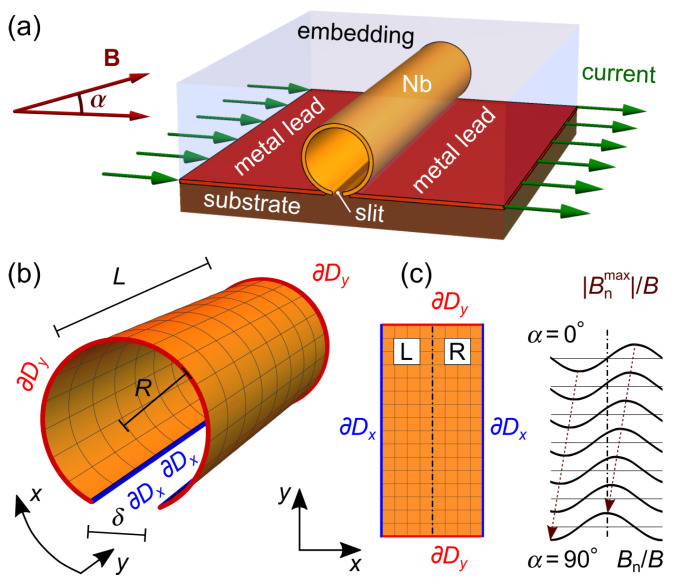
Geometry (**a**) and the mathematical model (**b**) of an open nanotube. (**c**) Unwrapped view of the nanotube surface. Normally conducting current leads are attached to the slit banks and correspond to the $\partial D_{x}$ boundaries. The evolution of the location of the $|B_{n}|$ maxima with increase in the magnetic field tilt angle α is also indicated.

An extension of a planar film into 3D brings about an inhomogeneity [14,15,16,17,18,19,20,21,22,23,24,25,26,27] of the normal-to-the-surface component $|B_{n}|$ of the applied magnetic field of induction B, such that vortices nucleate and move in the regions where $|B_{n}|$ is or close to maximal. In this regard, of particular interest are open superconductor nanotubes (that is, nanotubes with a slit), see Figure 1, in which, under an azimuthal transport current, vortices are constrained to move within the half-tubes and vortex jets are *non-expanding* [28]. Previously, the correlated dynamics of vortices in open nanotubes were investigated numerically with foci on ac drives [29] and transient regimes [30]. These predictions can be examined for, e.g., open Nb nanotubes fabricated by the self-rolling technology [31,32,33]. However, while the average induced voltage *U* and its frequency spectrum $f_{U}$ contain information on the vortex arrangements [28], the effects of magnetic-field tilting on their stability and the transitions between these arrangements have not been investigated so far. At the same time, it is known [34] that by tilting the direction of an external magnetic field with respect to the plane of a thin-film superconductor, the dynamics of vortices and the associated voltage responses can be substantially modified.

Here, we present an approach for the steering of vortices in open superconductor nanotubes by tilting the vector B at an angle α in the plane perpendicular to the nanotube axis. The numerical modeling is based on the time-dependent Ginzburg–Landau (TDGL) equation. Distinct from $\alpha =0^{\circ }$, when vortices move paraxially in opposite directions within each half-tube, an increase in α displaces the areas with close-to-maximum $|B_{n}|$ to the close(opposite)-to-slit regions, giving rise to descending (ascending) branches in $f_{U}\left(\alpha \right)$. At lower *B*, a critical angle $\alpha_{c}$ is revealed, at which the close-to-slit vortex chains disappear and $f_{U}$ evolves to the $nf_{1}$-type [n≥1: an integer, $f_{1}$: vortex nucleation frequency]. At higher *B*, $f_{U}$ is largely blurry due to multifurcations of the vortex trajectories in the opposite-to-slit vortex jet moving in the reverse direction with respect to the close-to-slit vortex chains. In all, our findings have implications for the tuning of GHz-frequency spectra in microwave applications and on-demand steering of vortices as information carriers.

## 2. Results

The studied geometry is shown in Figure 1a. An open superconductor nanotube of length L=5 µm and radius R=390 nm, with a slit of width δ=60 nm, is exposed to an azimuthal transport current of density $j_{\mathrm{tr}}$. A magnetic field of induction B is applied perpendicular to the tube axis at an angle α relative to the substrate plane, varying between $0^{\circ }$ and $90^{\circ }$. Under the action of the transport current, vortices nucleate at the free edges [boundaries $\partial D_{y}$ in Figure 1b] of the tube, move along the tube axis, and denucleate at the opposite free edges. At $\alpha =0^{\circ }$, the vortices in the opposite half-tubes move in reverse directions due to the sign reversal of $B_{n}$, see Figure 1c. In what follows, we refer to the vortex arrangements and the half-tubes as “R” (right) and “L” (left), see Figure 1c. With an increase in α, the R maximum of $B_{n}$ shifts towards the opposite-to-slit region, while the L maximum of $|B_{n}|$ shifts towards the L slit bank. This continues until two $|B_{n}|$ maxima occur just at the slit banks outside of the nanotube and the $B_{n}$ maximum coincides with the middle of the nanotube surface at $\alpha =90^{\circ }$.

The TDGL equation was solved for parameters typical for Nb films [35] and a film thickness of d=50 nm, resulting in a current density of 1 GA/m^2^ corresponding to a transport current of 0.25 mA. Details on the equations and boundary conditions are provided elsewhere [28]. Modeling was performed for $j_{\mathrm{tr}}=16$ GA/m^2^ at a temperature $T/T_{c}=0.952$, where $T_{c}$ is the superconducting transition temperature. The temperature $T/T_{c}=0.952$ is chosen as a representative case. For temperatures sufficiently lower than the critical one, the coherence length and the vortex core radius are significantly smaller, which decreases the effects of vortex confinement in the nanotube. For temperatures still closer to the critical one, the superconductivity is substantially weakened.

**Figure 2:**
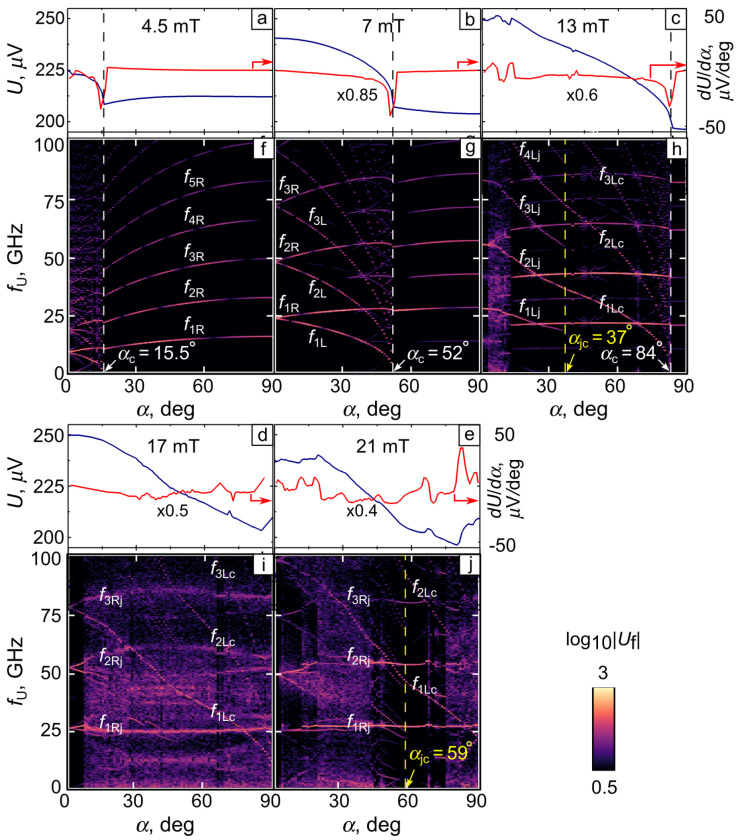
(**a**–**e**) Average induced voltage *U* and (**f**–**j**) its frequency spectrum $f_{U}$ as functions of the magnetic field tilt angle α for a nanotube with R=390 nm at the transport current density 16 GA/m^2^.

Figure 2 presents the average induced voltage *U*, its derivative with respect to the magnetic field tilt angle α, and the induced-voltage frequency spectrum $f_{U}$ as functions of α for a series of magnetic field values. At lower fields (4.5–13 mT), as α increases, $f_{U}\left(\alpha \right)$ exhibits an abrupt transition from a regime with crossing ascending and descending branches to a regime of $nf_{1}$-harmonics of the vortex nucleation frequency $f_{1}$. The transitions occur at some critical angle $\alpha_{c}$, which increases with an increase in *B*. At the same time, U(α) manifests a sharp bend at $\alpha ≲\alpha_{c}$, while dU/dα exhibits a minimum at $\alpha_{c}$. While $f_{U}\left(\alpha \right)$ decreases for $\alpha <\alpha_{c}$, it slowly increases for $\alpha >\alpha_{c}$ at ≲7 mT, and it is almost constant for ≳7 mT. At higher fields (17–21 mT), $f_{U}$ is blurry, though one can observe constant-frequency and descending branches. Overall, U(α) decreases by about 20% as α increases from $0^{\circ }$ to $90^{\circ }$.

**Figure 3:**
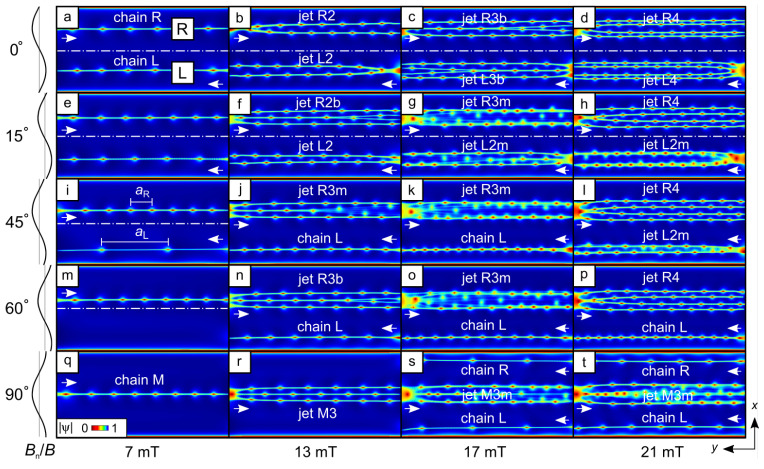
Snapshots of the absolute value of the superconducting order parameter |ψ| overlaid with the accumulated vortex paths for the nanotube with R=390 nm at the transport current density $j_{\mathrm{tr}}=16$ GA/m^2^. Evolution of vortex chains to vortex jets at $\alpha =0^{\circ }$ (**a**–**d**), $\alpha =15^{\circ }$ (**e**–**h**), $\alpha =45^{\circ }$ (**i**–**l**), $\alpha =60^{\circ }$ (**m**–**p**), and $\alpha =90^{\circ }$ (**q**–**t**). L: left; R: right (half-tube); M: middle; b: bifurcations; m: multifurcations. The number in the jet name corresponds to the number of vortex chains in the jet. The direction of the vortex motion is indicated by the arrows. The dash-dotted lines are the midlines.

Further insights into the features of $f_{U}\left(\alpha \right)$ can be gained from an analysis of the spatial evolution of the absolute value of the superconducting order parameter |ψ| as functions of α and *B*. Indeed, the evolution of *U* and $f_{U}$ at 4.5 mT and 7 mT can be understood with the aid of the snapshots of |ψ|(x,y) overlaid with the accumulated vortex trajectories in Figure 3. Thus, at 7 mT and $\alpha =0^{\circ }$, the vortices are arranged in two chains located symmetrically relative to the (dash-dotted) midline. At $\alpha =0^{\circ }$, the intervortex distances in the L and R chains are equal, $a_{L}=a_{R}$. A tilt in B of $15^{\circ }$ leads to the inequality $a_{R}<a_{L}$, which becomes more pronounced with $a_{L}≃3a_{R}$ at $\alpha =45^{\circ }$. While the vortex velocities in the R and L half-tubes remain almost equal to each other, the vortex nucleation frequencies in the R and L half-tubes differ by a factor of $f_{R}/f_{L}≃3$. This ratio agrees well with the frequency ratio $f_{1}R/f_{1}L$ at $\alpha =45^{\circ }$ in Figure 2g. Here, and in what follows, we use the subscripts “R” and “L” to refer to the frequencies associated with the vortex arrangements in the right and left half-tubes [as illustrated in Figure 1c], respectively.

At 7 mT, upon reaching the critical angle $\alpha_{c}=52^{\circ }$, the L vortex chain disappears, decisively affecting the spectrum $f_{U}$. Namely, for angles $\left(\alpha_{c}-\alpha \right)/\alpha_{c}\ll 1$, the branches $nf_{1}L$ descend very rapidly and U(α) exhibits a fast decrease, see Figure 2b. For $\alpha >\alpha_{c}$, the spectrum is of the $nf_{1}R$ type. Herein, $f_{1}R$ is associated with the harmonics of the vortex nucleation frequency in the single L chain, which is displaced to the opposite-to-slit area (midline), see Figure 3q. The vortex arrangements at 4.5 mT are qualitatively similar, but differ by a smaller number of vortices and a smaller $\alpha_{c}$.

At 13 mT and for $\alpha =0^{\circ }$, there are two vortex jets, each consisting of two vortex chains and moving within each half-tube. At $3^{\circ }≲\alpha ≲13^{\circ }$, the R chain exhibits multifurcations of vortex trajectories and turns into a jet of three vortex chains, Figure 3f. The multifurcations result in a blurry $f_{U}$, Figure 2h. Upon reaching the angle $\alpha_{\mathrm{jc}}=37^{\circ }$, the L jet turns into a vortex chain, Figure 3j. Accordingly, $f_{U}$ evolves from the $nf_{1}\mathrm{Lj}$ type to the $nf_{1}\mathrm{Lc}$ type, Figure 2h, with $f_{1}\mathrm{Lc}/f_{1}\mathrm{Lj}\approx 2$ at $\alpha_{\mathrm{jc}}=37^{\circ }$. Here, and in what follows, the subscripts “j” and “c” denote that the (descending with an increase in α) frequency branches in the voltage spectra are related to a *vortex jet* and a *vortex chain* in the left half-tube, respectively. The descending branches $nf_{1}\mathrm{Lc}$ reach zero at $\alpha_{c}=84^{\circ }$, pointing to the absence of an L chain at larger values of α, Figure 3r.

At 17 mT (21 mT) and for $\alpha =0^{\circ }$, the jets consist of three (four) chains of vortices, respectively. Their evolution with an increase in α can be outlined as follows. The R jet is continuously shifted to the opposite-to-slit region. At 17 mT, multifurcations of the vortex trajectories are more pronounced, which makes $f_{U}$ more blurred. One can recognize a few descending branches in $f_{U}$ in Figure 2i, which are due to a reduction in the number of vortices in the L chain as it approaches the L slit bank. It is noteworthy that for both fields, at $\alpha =90^{\circ }$, a vortex chain appears in the R half-tube, making the vortex pattern symmetric relative to the midline. At 21 mT, a transition from the L jet to the L chain occurs at $\alpha_{\mathrm{jc}}=59^{\circ }$, Figure 3p. In this way, with an increase in α from $0^{\circ }$ to $90^{\circ }$, vortices can be steered to any point of the nanotube, while features in $f_{U}$ can be attributed to particular vortex configurations and the transitions between them. It is interesting to note that with an increase in α, a transition is realized from a vortex jet to a vortex chain, which does not occur in planar thin films at moderately strong currents (i.e., when non-equilibrium effects can be ignored).

## 3. Methods

Numerical modeling was performed by adopting the procedure described in ref. [28] to the geometry used here, in which the direction of the applied magnetic field was varied with respect to the substrate plane. Namely, an open nanotube in a homogeneous perpendicular-to-tube-axis magnetic field was mapped to a planar membrane in a modulated out-of-plane field, see Figure 1. The mathematical model of the nanotube is represented by a 2D surface denoted as *D* and parameterized by orthonormal coordinates *x* and *y*, along with a normal unit vector n. Surface *D* is embedded in a 3D space with Cartesian coordinates *X*, *Y*, and *Z*. Modeling was based on a numerical solution of the 2D TDGL equation, which, in its dimensionless form, is:

(1) $$\left(\partial_{t}+i\varphi \right)\psi ={\left(\nabla -iA\right)}^{2}{\psi +(1-|\psi |}^{2})\psi .$$

Here, $\partial_{t}$ denotes the derivative with respect to time *t*, ∇ is a 2D nabla operator on the surface, **A** is the vector potential, the scalar potential φ determines the electric field E=−∇φ, and ψ≡ψ(x,y,t) is the complex superconducting order parameter, which depends on the coordinates *x* and *y* and evolves with time *t*. Using the gauge freedom of the vector potential A, its normal component can be chosen as $A_{n}=0$ in the vicinity of the nanotube surface *D*, and its normal derivative follows $\nabla_{n}A_{n}=0$ at *D*, where the subscript *n* denotes the projection of the corresponding vector onto the normal vector n (see, e. g., [36]). Being defined in the entire 3D space, the vector potential determines the magnetic induction $B=[\nabla_{3D}\times A]$, where $\nabla_{3D}$ is the nabla operator in the 3D space. The units for the dimensionless quantities in Equations (1)–(4) are provided in Table 1.

The superconducting current density is determined by jsc=Im(ψ*(∇−iA)ψ). The effects of the magnetic field induced by the superconducting currents are neglected. The applicability of this approximation is discussed elsewhere [30].

The Poisson equation for the scalar potential φ follows from the continuity of the total current density, which is given by the sum of the superconducting $j_{\mathrm{sc}}$ and normal $j_{n}$ components

(2) $$\Delta \varphi =\frac{1}{\sigma }\nabla \;\cdot \;j_{\mathrm{sc}},\;j_{n}=-\sigma \nabla \varphi .$$

The boundary conditions [37] for the TDGL are

(3) $${\left.\left(\partial_{y}-iA_{y}\right)\psi \right|}_{\partial D_{y}}=0,\;{\left.\psi \right|}_{\partial D_{x}}=0,$$

where $\partial D_{x}$ and $\partial D_{y}$ are the boundaries corresponding to the ends of the intervals for *x* and *y*, respectively, see Figure 1c. The transport current density $j_{\mathrm{tr}}=j_{\mathrm{tr}}e_{x}$ is introduced through the boundary conditions for the scalar potential

(4) $${\left.\partial_{y}\varphi \right|}_{\partial D_{y}}=0,\;{\left.\partial_{x}\varphi \right|}_{\partial D_{x}}=-j_{\mathrm{tr}}/\sigma .$$

The scalar potential is split into two terms, namely the non-divergent potential $\varphi_{\mathrm{ndiv}}$ and the induced potential $\varphi_{\mathrm{ind}}$. This separation allows for a faster convergence of the numerical algorithm [30,38] used for solving the Poisson equation.

As gauge-dependent numerical schemes may introduce enormous errors [39], link variables [30] were used for both the vector potential A (conjugated with coordinates) and the scalar potential φ (conjugated with time). The voltage between the leads was calculated as the difference in the scalar potentials averaged over the lead length

(5) $$U\left(t\right)=\frac{1}{L}\int_{0}^{L}dy\left(\varphi (t,W,y)-\varphi (t,0,y)\right).$$

Modeling was performed for parameters typical for Nb structures, see Table 2. For the nanotube with R=390 nm, a grid of 192×384 (x×y) points and a time step of Δt=0.025 ps were used. For other sizes and structures, the number of points was chosen to result in approximately the same density of grid points per unit length. An iterative method of solving the Poisson equation was used until the absolute value of the difference between the left and right sides of Equation (2) became smaller than 0.004 for all grid points.

More details on the applicability of the mathematical model, the link variables, and the splitting of the electric potential and further numerical details are given elsewhere [30].

## 4. Discussion

The major findings can be summarized as follows. First, the symmetry break associated with an increase in α leads to an increasingly stronger constraint of vortices in the L half-tube, while the primary effect of the magnetic field tilting on the vortices in the R half-tube consists of their displacement towards the opposite-to-slit area. Accordingly, the frequency of vortex nucleation in the R half-tube remains almost constant, and this is reflected in the very slowly increasing or almost constant-frequency branches in $f_{U}$. Second, the vortex nucleation frequency in the L half-tube decreases with an increase in α. At lower magnetic fields (4.5–13 mT), this leads to the disappearance of the L vortex chain at the critical angle $\alpha_{c}\left(B\right)$. At moderate and higher fields (4.5–13 mT), when the initial configuration of vortices at $\alpha =0^{\circ }$ evolves into vortex jets in both half-tubes, there is a transition from the L vortex jet to the L vortex chain at the transition angle $\alpha_{\mathrm{jc}}$. Both angles $\alpha_{c}$ and $\alpha_{\mathrm{jc}}$ increase with rising *B*. Third, the transition from a vortex jet to a vortex chain or the change in the number of vortex chains forming a vortex jet may be not abrupt, but may occur as a consequence of bi- or multifurcations of the vortex trajectories. In this case, the frequency spectrum $f_{U}$ is blurry, which complicates the identification of frequencies. Fourth, while a *unidirectional* vortex motion via a single vortex chain or vortex jet in the opposite-to-slit region is revealed for lower fields, a *bidirectional* vortex motion mediated by a centrally located vortex jet and two vortex chains at the slit banks is predicted for higher magnetic fields.

**Figure 4:**
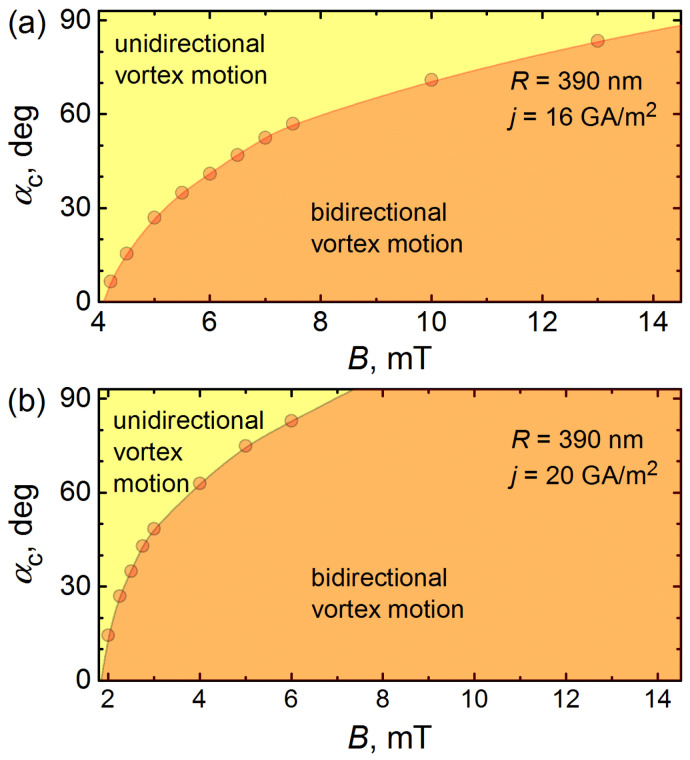
The critical angle $\alpha_{c}$ as a function of the magnetic field at $j_{\mathrm{tr}}=16$ GA/m^2^ (**a**) and $j_{\mathrm{tr}}=20$ GA/m^2^ (**b**), demarcating the regimes of unidirectional and bidirectional vortex motion.

The regimes of the unidirectional and bidirectional vortex motion are shown in Figure 4 and demarcated by the dependences $\alpha_{c}\left(B\right)$ for two current densities. The shape of $\alpha_{c}\left(B\right)$ at $j_{\mathrm{tr}}=16$ GA/m^2^ [see Figure 4a] implies that the highest sensitivity of the nanotube to the magnetic-field direction is achieved at about 4.25 mT. This magnetic field corresponds to the first occurrence of vortices in the nanotube, which makes it very sensitive to the magnetic field direction. With am increase in $j_{\mathrm{tr}}$ to 20 GA/m^2^ [see Figure 4b], the boundary between the unidirectional and bidirectional vortex motion regimes shifts towards lower magnetic fields.

We note that the phase boundary between the unidirectional and bidirectional vortex motion, in general, may be affected by the self-field $B_{\mathrm{self}}$. In our case, an approximate estimate for the self-field can be made by considering the tube generatrix as a thin film of thickness d=50 nm and width L=5μm using a 20 GA/m^2^ current density that corresponds to a current of 5 mA. The estimate $B_{\mathrm{self}}=\mu_{0}I/2\pi L\ln\left(2L/d\right)≲1$ mT means that the phase diagram in Figure 4a is expected to be almost unaffected, while there might be a minor effect for the phase boundary in Figure 4b due to the $B_{\mathrm{self}}$ correction to the applied magnetic field.

## 5. Conclusions

Finally, we would like to comment on a possible experimental examination and the general relevance of the obtained results. Deviations in B by an angle α of a few degrees from the perpendicular-to-tube axis and parallel-to-substrate direction may easily occur in experiments [33], so transitions in $f_{U}$ could be useful for checking the sample/field alignment. In addition, the evolution of vortex arrangements with an increase in α is interesting from the viewpoint of both basic research [41,42] and emerging functionalities. For the open nanotubes studied here, the steering of vortex arrays to desired parts of the nanotube via magnetic field tilting and vortex-jet-to-vortex-chain transitions, which does not occur in planar thin films at moderately strong transport currents [13], is interesting. Conceptually, the steering of vortex chains, vortex jets, and more complex arrangements is similar to using a magnetic field for controlling the vortex dynamics [43], but it surpasses vortex guiding in nanoengineered pinning landscapes in terms of reconfigurability [44,45,46,47]. While spatio- and time-resolved studies of vortex arrangements in 3D nanoarchitectures are challenging, deducing their properties from features of the global observables *U* and $f_{U}$ represents a viable approach to this end.

## Figures and Tables

**Table 1:** Dimensional units for quantities in Equations (1)–(4).

Parameter	Unit	Value of Nb at $T/T_{c}=0.952$
Time	$\xi^{2}/D$	2.8 ps
Length	ξ	60 nm
Magnetic field	$\Phi_{0}/2\pi \xi^{2}$	92 mT
Current density	$\hbar c^{2}/8\pi \lambda^{2}\xi e$	60 GA $m^{-2}$
Electric potential	$\sqrt{2}H_{c}\xi \lambda /c\tau$	111 µV
Conductivity	$c^{2}/4\pi \kappa^{2}D$	31 (µ${\Omega \;m)}^{-1}$

**Table 2:** Material parameters used in the simulations. $T_{c}$: superconducting transition temperature; $v_{F}$: Fermi velocity; $m_{e}$: electron mass.

Parameter	Denotation	Value for Nb
Electron mean free path	*l*	6 nm
Fermi velocity	$v_{F}=\sqrt{2E_{F}/m_{e}}$	600 km/s
Diffusion coefficient	$D=lv_{F}/3$	12 cm^2^/s
Normal conductivity [35,40]	$\sigma =l/[3.72\times 10^{-16}\;\Omega \;m^{2}]$	16 (µ${\Omega \;m)}^{-1}$
Relative temperature	$T/T_{c}$	0.952
Penetration depth	$\lambda =\lambda_{0}\sqrt{\xi_{0}/\left(2.66l\left(1-T/T_{c}\right)\right)}$	278 nm
Coherence length	$\xi =0.855\sqrt{\xi_{0}l/\left(1-T/T_{c}\right)}$	60 nm
GL parameter	κ=λ/ξ	4.7

## Data Availability

The data presented in this study are available on reasonable request from the corresponding author.
